# Comparison of SARIMA model, Holt-winters model and ETS model in predicting the incidence of foodborne disease

**DOI:** 10.1186/s12879-023-08799-4

**Published:** 2023-11-16

**Authors:** Xiaobing Xian, Liang Wang, Xiaohua Wu, Xiaoqing Tang, Xingpeng Zhai, Rong Yu, Linhan Qu, Mengliang Ye

**Affiliations:** 1 College of Public Health, Chongqing Medical University, Chongqing, China; 2 Nan’an District Center for Disease Control and Prevention, Chongqing, China; 3 School of Traditional Chinese Medicine, Chongqing Medical University, ChongQing, China; 4 School of The First Clinical College, Chongqing Medical University, ChongQing, China

**Keywords:** Foodborne Disease, SARIMA model, Holt-Winters model, Exponential smoothing model

## Abstract

**Background:**

According to the World Health Organization, foodborne disease is a significant public health issue. We will choose the best model to predict foodborne disease by comparison, to provide evidence for government policies to prevent foodborne illness.

**Methods:**

The foodborne disease monthly incidence data from June 2017 to April 2022 were obtained from the Chongqing Nan’an District Center for Disease Prevention and Control. Data from June 2017 to June 2021 were used to train the model, and the last 10 months of incidence were used for prediction and validation The incidence was fitted using the seasonal autoregressive integrated moving average (SARIMA) model, Holt-Winters model and Exponential Smoothing (ETS) model. Besides, we used MSE, MAE, RMSE to determine which model fits better.

**Results:**

During June 2017 to April 2022, the incidence of foodborne disease showed seasonal changes, the months with the highest incidence are June to November. The optimal model of SARIMA is SARIMA (1,0,0) (1,1,0)_12_. The MSE, MAE, RMSE of the Holt-Winters model are 8.78, 2.33 and 2.96 respectively, which less than those of the SARIMA and ETS model, and its prediction curve is closer to the true value. The optimal model has good predictive performance.

**Conclusion:**

Based on the results, Holt-Winters model produces better prediction accuracy of the model.

## Introduction

Foodborne disease (FBD) are a major cause of morbidity and mortality and a very important public health problem worldwide. According to the estimation of World Health Organization (WHO), 600 million people worldwide fall ill each year due to the consumption of contaminated food, resulting in 420,000 deaths and a loss of 33 million healthy lives [1, 2]. This equates to 550 disability-adjusted life years (DALYs). A DALYs can be considered as a loss of health and life for one year [3, 4], WHO FBD Epidemiology Reference Group (FERG) estimated that FBD cause a global loss of 33 million DALYs annually [5], which will have a huge impact on people’s lives and health. These effects are not limited to low-income and high-income countries, such as those in Europe, where 41 to 49 DALYs per 100,000 population can be attributed to FBD [5]. Food insecurity costs $110 billion a year in lost productivity and health care costs among low-and middle-income countries [6]. As the world’s largest developing country, the situation of FBD in China is also not optimistic. An analysis of nearly 2,500 Chinese articles on diseases from 1994 to 2005 revealed 1,082 cases of bacterial FBD, if relying solely on these numbers alone would seriously underestimate the number of FBD in China, and a national acute gastroenteritis survey estimates that 748 million cases of acute gastroenteritis and 420 million medical consultations occur in China every year [7]. Patients with FBD often lack awareness of the severity of the disease, which may cause them to postpone medical treatment. This delay can easily cover up the outbreak of food safety incidents, which is not conducive to the timely control of disease outbreak. These health effects have economic implications for affected people, healthcare systems, food producers and distributors [8]. To address these issues, China has begun using systems such as Pulse Net domestically to track and correlate food-borne pathogens.

FBD ranges from mild self-limiting diseases to life-threatening food poisoning [9], and foodborne pollutants are abundant. They include viruses and bacteria, parasites, chemicals, toxins and allergens that cause a wide variety of diseases [5]. In addition, many foodborne hazards are transmitted by other means: through water, soil or air; by direct contact between people, or between people and animals. FBD are becoming a greater challenge due to new and emerging microorganisms and toxins, the growth of antibiotic resistance, and increasing food contamination due to new environmental and food production methods [10]. The effort of improving food safety and reducing the burden of FBD relies on data from FBD monitoring and epidemic investigations to help prioritize food safety interventions, policies, and practices [11]. Recognizing the necessity for global and regional estimates of FBD to guide public health policy, the WHO launched the Estimating the Global Burden of FBD Initiative in 2006. The main aim of the initiative is to get policy makers and some people involved in food safety to set up appropriate evidence-based regulations, which can also improve the capacity of countries to assess their FBD burden. In addition, since 2011, China has established a web-based FBD monitoring platform, which has gradually played a role in the early warning of food safety emergencies, food safety emergencies, and research on the burden brought by FBD [12]. Through these monitoring platforms, we can timely discover clusters, improve the early identification, warning, and prevention and control capabilities of food safety risks, and grasp the baseline of important FBD.

According to WHO estimates, approximately 2.2 million people worldwide die each year due to foodborne or waterborne diarrhea. There are approximately 600 million cases of foodborne diseases worldwide each year, with a death toll of many people, of which 125,000 are children under the age of 5 [13]. The Centers for Disease Control reported that 48 million people in the United States get sick from FBD each year, with 128,000 hospitalized and 3,000 dying [14]. Nan’an District is one of the main urban areas of Chongqing, which is an area driven by light industry, catering and tourism industry, and also with many people who suffer from FBD here. However, due to the low sensitivity of FBD surveillance, there is a certain gap between the number of cases reported and the actual situation, so it is necessary to choose a better prediction model for FBD.

The autoregressive integrated moving average (ARIMA) model is a widely used time series analysis tool, which is widely used to predict infectious diseases such as malaria, hemorrhagic fevers, hand, foot and mouth disease, influenza, COVID -19 and tuberculosis [15]. Additionally, ARIMA-related hybrid models such as Seasonal Autoregressive Integrated Moving Average (SARIMA) was also developed as modeling candidates for future trend prediction. SARIMA model and Holt-Winters model are two of the most widely used time series forecasting methods, which are suitable for different types of time series models and can reflect time changes as well as periodic changes in the original data [16]. Exponential Smoothing (ETS) model is also widely used in predicting infectious disease, such as brucellosis and epidemiological surveillance [17].

Previous studies on FBD mainly focused on analyzing public surveillance data and estimating the actual incidence rate of FBD in a country or a region, and assess the disease burden caused by various pathogens [18]. However, there are few studies that compare the advantages and disadvantages of the three models. This study proposes to establish a SARIMA model, Holt-Winters and ETS model by the number of monthly incidences and compare the advantages and disadvantages of three different models, so as to select the optimal model.

## Materials & methods

### Data source

China’s FBD surveillance platform was established in 2011, which mainly includes: the FBD Outbreaks Surveillance System, the FBD Surveillance and Reporting System, the National Molecular Traceability Network for FBD and other surveillance systems. The China National Center for Food Safety Risk Assessment maintains and manages the platform for data collection and periodic reporting to the National Health Commission [20]. The data of the FDB and population used in this study were obtained from Chongqing Nan’an District Center for Disease Prevention and Control from June 2017 to April 2022. FBD data were collected from 25 medical institutions for monitoring sites and 14 primary medical institutions for health emergency response teams in Nan’an District, and reporting form of the data is the incidence of cases. We collect continuous monthly data, which helps input and build the model. Data from June 2017 to June 2021 were used to train the model, and the last 10 months of incidence were used for prediction and validation. The incidence was fitted using the SARIMA model, Holt-Winters model and ETS model.

### Data processing and analysis

SPSS 25.0 was used for data preprocessing and descriptive statistics, the SARIMA, Holt-Winters and ETS models were developed by R 4.1.2. In addition, all the figures are also made of R 4.1.2. In this study, p < 0.05 was considered statistically significant.

### SARIMA model

The ARIMA model consists of three parts: autoregression order (p), difference order (d), and moving average order (q) [21]. The SARIMA model is a Seasonal ARIMA, which consists by seasonal effect, long-term trend effect, periodic change and random disturbance. The general form of the SARIMA model is (p, d, q) × (P, D, Q) s, p, d and q are non-negative integers, representing the order of non-seasonal autoregressive (AR) term, non-seasonal difference and non-seasonal moving average (MA), respectively. P, D and Q are also non-negative integers indicating the order of seasonal AR term, seasonal difference term and seasonal MA term respectively; S is the length of the seasonal period [22].

Generally speaking, time series modeling methods include the following three steps: model recognition, parameter estimation, and diagnostic checks. Firstly, if necessary, perform appropriate differencing on the sequence to achieve stationarity and normality. We use the augmented Dickey Fuller (ADF) unit root test to estimate whether the time series is stationary, if result of the ADF test is significant, the sequence is proven to be stationary. Secondly, the time dependent structure of the transformed data is identified by examining the autocorrelation functions (ACF) and partial autocorrelation functions (PACF) of the transformed data [23]. Besides, the values of p, d and q, q are finally determined by considering the smallest Akaike information criterion (AIC) and Bayesian information criterion (BIC) values corresponding to the higher prediction accuracy. At last, in order to test the normality of SARIMA residuals, the Ljung-Box Q test was used to diagnose whether the residual error sequence was a white-noise sequence [24].

### Holt-winters model

The component form of the Holt-Winters model consists of four equations, namely the prediction equation and three smoothing equations [25]. The characteristic of Holt-Winters model is to eliminate some random fluctuations while correcting seasonal trends. It assigns different weights to data from each period and reasonably predicts future development trends. The α (level) and β (slope) of the trend should be between 0 and 1, and when a value close to 0 means that the estimation of current/future time points is based on recent observations [26].

### ETS model

ETS model take the errors, trends, and seasonal components of a given time series into consideration, and evaluates possible alternative models before selecting the best performing model to simulate the data. ETS model considered comprehensive historical information, it has three main parameters: error, trend, and seasonal component, which can be additive (A), multiplicative (M), or none (N). The ETS method includes several detailed methods, such as single ETS, double ETS, Holt trend ETS (with or without seasonal features), and other methods based on various features of the original sequence. The optimal model is selected according to the AIC minimum, the corrected Akaike message criterion (AICc), or the BIC [27]. What’s more, Ljung-Box Q test was used to diagnose whether the residual sequence is white noise sequence.

### Evaluation metrics

In order to evaluate the performance of the SARIMA, Holt-Winters and ETS model, we tested the fitting values. Several performance indexes, namely, root means square error (RMSE), mean absolute error (MAE), mean absolute percentage error (MAPE) and means square error (MSE) are used to determine the predictive efficiency of the three models [28]. Many researchers have used these metrics to assess the accuracy of models, when the MSE, MAE, and RMSE values of the model are smaller, the fitting degree of the model is better. If all three indicators of a model are lower than another model, then the model is more superior. For the measure of these metrics, the smallest value corresponds to the best method. The following are the calculation methods for some indicators.

$${\rm RMSE} = \sqrt{\sum {(actual-forecast)}^{2}\times (1/ sample size)}$$

$${\rm MAE} = \sum \left(\left|actual-forecast\right|\right)\times (1/ sample\, size)$$

$${\rm MAPE} = (1/ {\rm sample\, size} ) \times \sum \begin{array}{c}\left[\frac{\left|actual-forcast\right|}{\left|actual\right|}\right]\\ \end{array} \times 100 \%$$

$${\rm MSE} = \sum {(actual-forecast)}^{2}\times (1/ sample\, size)$$

### Ethics approval and consent to participate

The research protocols and informed consent forms submitted for this project comply with the principles of medical ethics and the requirements of the Declaration of Helsinki. This study was approved by the Ethics Committee of Nan’an District for center disease control and prevention, and informed consent was obtain from all the participant. All the data collection in this study are was in accordance to the Law of the People’s Republic of China on the Prevention and Treatment of Infectious Diseases.

## Results

### Descriptive statistics

Table 1 shows the incidence rate of each month from June 2017 to April 2022 based on the average population of the Nan’an District. The month with the highest incidence was November 2021, which reached 17.03 per 100,000, while the lowest incidence occurred in January 2019 with 0.08 per 100,000.

**Table 1:** Distribution of FBD incidence in Nan’an District, Chongqing, 2017 to 2022

Year	Month (incidence rate/hundred thousand)											
	1	2	3	4	5	6	7	8	9	10	11	12
2017						53(4.72)	29(2.58)	45(4.01)	72(6.42)	21(1.87)	34(3.03)	8(0.71)
2018	5(0.44)	8(0.7)	6(0.52)	9(0.78)	24(2.09)	19(1.66)	45(3.92)	22(1.92)	40(3.49)	38(3.31)	48(4.18)	10(0.87)
2019	1(0.08)	6(0.51)	15(1.27)	13(1.1)	41(3.48)	121(10.28)	89(7.56)	76(6.46)	59(5.01)	116(9.86)	181(15.38)	14(1.19)
2020	3(0.25)	7(0.58)	9(0.75)	26(2.17)	77(6.42)	112(9.33)	117(9.75)	91(7.58)	92(7.67)	93(7.75)	155(12.92)	28(2.33)
2021	19(1.58)	20(1.66)	30(2.49)	31(2.57)	93(7.72)	54(4.49)	86(7.14)	93(7.72)	200(16.61)	184(15.28)	205(17.03)	60(4.98)
2022	13(1.08)	18(1.49)	45(3.73)	26(2.15)								

### The analysis of SARIMA model results

**Fig. 1:**
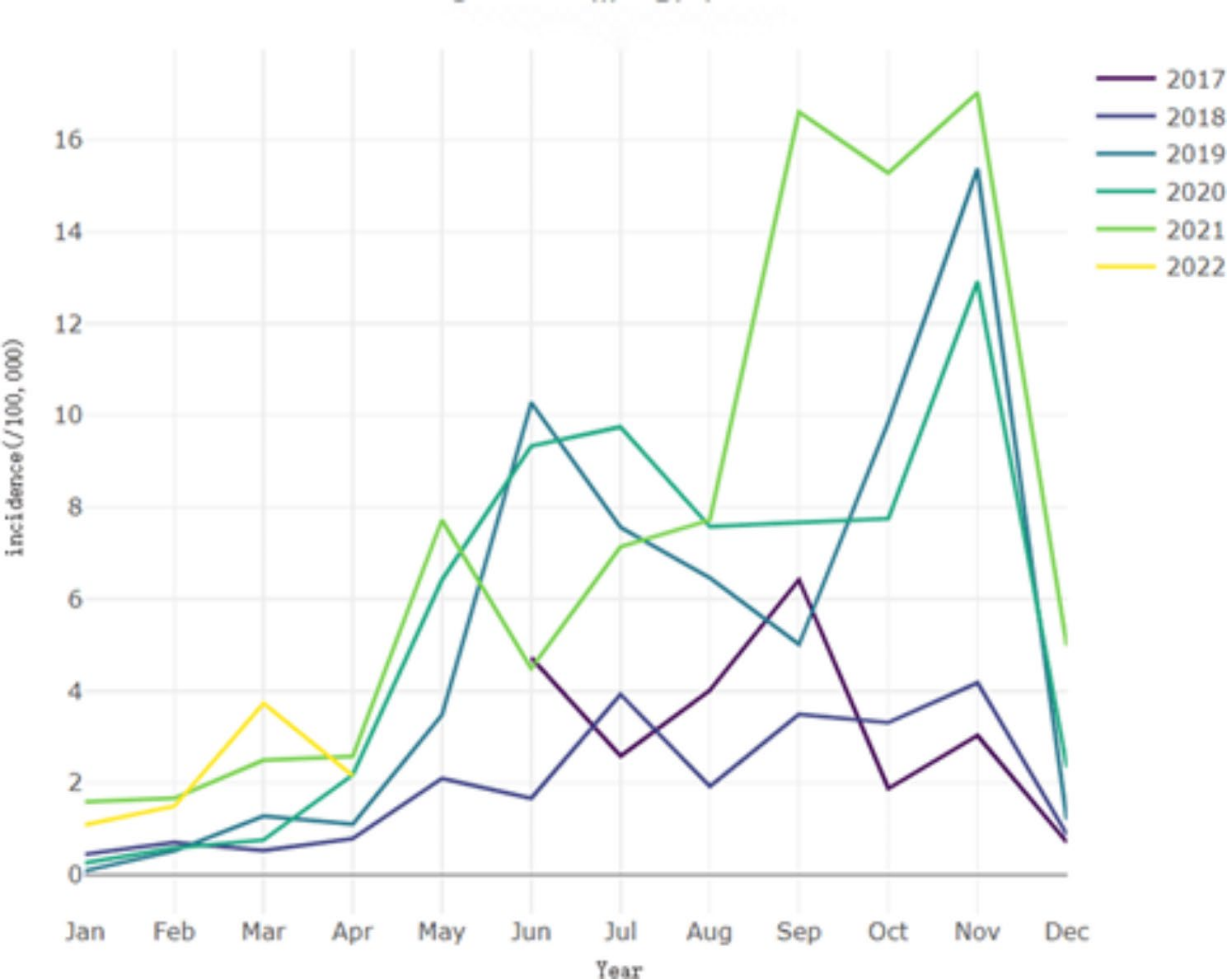
Seasonal distribution of FBD in Nan’an District

This study used the “STL” function to decompose the sequence, Fig. 1 presents seasonal distribution of FBD in Nan’an District from June 2017 to April 2022. It can be clearly seen from the figure that June to November is the peak period of incidence.

Only six models passed the residual test and parameter test, the six models were SARIMA (1,0,0) (1,1,0)_12_, SARIMA (0,0,1) (0,1,1)_12_, SARIMA (0,0,1) (0,1,0)_12_, SARIMA (0,0,1) (1,1,0)_12_, SARIMA (0,0,0) (0,1,1)_12_. The AIC values of the five candidate models are listed in Table 2, and we finally confirmed SARIMA (1,0,0) (1,1,0)_12_ model with drift is finally selected as the optimal SARIMA model after comparing the AIC values. Besides, the evaluation criteria for the SARIMA model are displayed in Table 3.

**Table 2:** AIC and BIC values for candidate SARIMA models

Candidate Models	AIC	BIC
SARIMA (1,0,0) (1,1,0)_12_ with drift	188.83	193.67
SARIMA (0,0,1) (0,1,1) _12_ with drift	189.79	194.62
SARIMA (0,0,1) (0,1,0) _12_ with drift	189.21	192.43
SARIMA (0,0,1) (1,1,0) _12_ with drift	190.23	195.06
SARIMA (0,0,0) (0,1,1) _12_ with drift	194.49	197.71

Best: SARIMA (1,0,0) (1,1,0) _12_ with drift

Note: SARIMA, Seasonal Autoregressive Integrated Moving Average; AIC, Akaike information criterion; BIC, Bayesian information criterion.

### The analysis of ETS model results

ETS programming functions were used to simulate time series data sets on the incidence of FBD, from which we found an appropriate ETS model (AIC = 228.24, BIC = 256.62). The evaluation criteria for the ETS model are displayed in Table 3.

### The analysis of Holt-winters model results

R software automatically selects the model that best fits the original data. The results showed that Holt-Winters model have good prediction accuracy of the model. Table 3 showed the evaluation index of the Holt-Winters model.

### Model comparison

Many scholars have conducted research on disease prediction models. SARIMA model is a model for relatively stable time series data. It integrates time trend, seasonal, periodic change, random error and other factors to quantify the model parameters [29]. However, the Holt-Winters model has a relatively simple principle, and has a high prediction accuracy for diseases with periodic regularity [30]. This method assigns different weights to the distance of data on the timeline, which is suitable for predicting individual time series data. We found MSE, MAE, RMSE of the Holt-Winters model are less than those of the SARIMA and ETS model Table 3. What’s more, the Holt-Winters model also has better predictive accuracy than SARIMA. This may be due to the characteristics of each model. In terms of models alone, the SARIMA model is more suitable for predicting data with stable changing trends than the Holt-Winters model, while the Holt-Winters model is more suitable for predicting data with single changing trends.

**Table 3:** Evaluation indicators of the three models

Evaluation indicators	ETS	Holt-Winters	SARIMA
MSE	14.45	8.78	17.88
MAE	2.68	2.33	2.75
RMSE	3.80	2.96	4.23
MAPE	29.42	39.29	28.80

Note: MSE, means square error; MAE, mean absolute error; RMSE, root means square error; MAPE, mean absolute percentage error; ETS, Exponential Smoothing; SARIMA, Seasonal Autoregressive Integrated Moving Average.

The forecast results from July 2021 to April 2022 of FBD incidence in Nan ‘an district according to the SARIMA (1, 0, 0) (1, 1, 0) _12_ model, ETS model and Holt-Winters model are shown in Table 4. The observed incidence of SARIMA (1, 0, 0) (1, 1, 0) _12_ model in September and October 2021, the observed incidence of ETS model in December 2021 and the observed incidence of Holt-Winters model in September, November and December 2021 were not within the 95% confidence interval (CI) of the predicted values.

**Table 4:** The prediction of SARIMA (1, 0, 0) (1, 1, 0) _12_ model, ETS model and Holt-Winters model

Month	Incidence	SARIMA model(95%CI)	ETS model(95%CI)	Holt-Winter
				model(95%CI)
2021/7/1	7.14	7.11(1.54,12.68)	8.48(-0.04,17)	13.09(12.51,13.67)
2021/8/1	7.72	6.36(0.25,12.47)	7.12(-0.53,14.76)	7.19(6.57,7.81)
2021/9/1	16.61	6.75(0.54,12.97)	8.74(-1.23,18.71)	11.63(10.61,12.65)
2021/10/1	15.28	7.9(1.66,14.14)	8.94(-1.83,19.71)	11.65(10.3,12.99)
2021/11/1	17.03	13.24(7,19.49)	11.86(-3.18,26.9)	15.19(13.03,17.35)
2021/12/1	4.98	2.09(-4.15,8.34)	1.75(-0.58,4.08)	2.46(1.74,3.17)
2022/1/1	1.08	1.33(-4.91,7.58)	0.94(-0.37,2.24)	1.43(0.74,2.12)
2022/2/1	1.49	1.47(-4.78,7.71)	1.15(-0.52,2.81)	2.37(1.18,3.56)
2022/3/1	3.73	2.19(-4.05,8.43)	2(-1.02,5.03)	2.4(0.86,3.94)

Note: ETS, Exponential Smoothing; SARIMA, Seasonal Autoregressive Integrated Moving Average; CI, confidence interval.

**Fig. 2:**
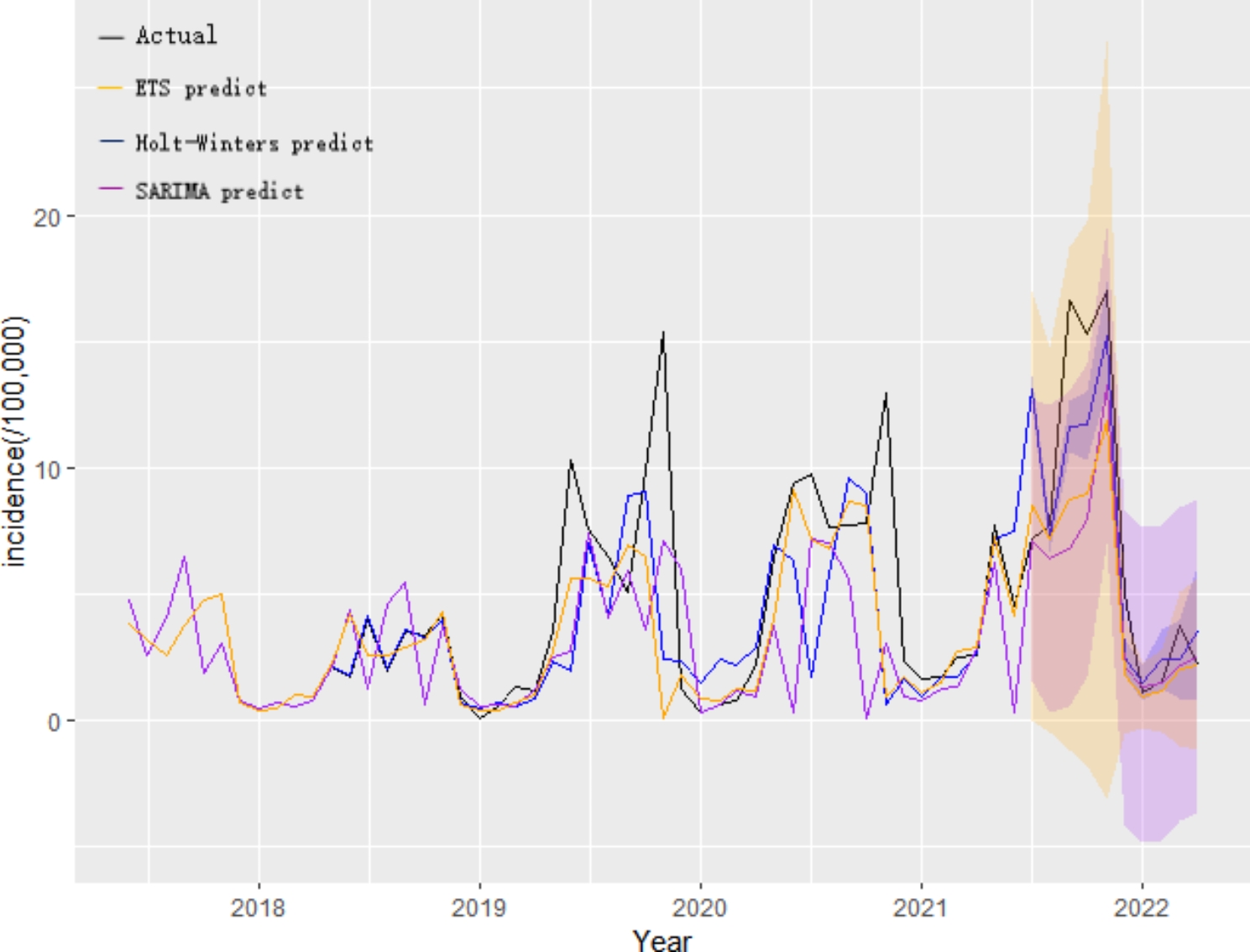
Fitting status between the actual incidence of foodborne illness in Chongqing Nan’an District from June 2017 to June 2021 and the predicted incidence from July 2021 to April 2022. The black line indicates the actual incidence rate, orange, blue, and purple indicate the incidence rates predicted by the ETS, Holt-Winters, and SARIMA models, and hazy areas indicate the upper and lower ranges of the 95% CI for the predicted incidence rates of foodborne illness

In addition, Table 5 shows the comparison of prediction results of three models, in most months of the forecast, the forecast error of the Holt-Winters model was smaller than that of the other two models. Comparing the fitting effects of the three models, it can be seen from Fig. 2 that the fitting value of the Holt-Winters model is closer to the actual value. Therefore, it can be concluded that the optimal model is Holt-Winters model.

**Table 5:** Comparison of prediction results of three models

Month	Incidence	Holt-Winters forecast		SARIMA forecast		ETS forecast	
		Forecast	Forecast error (%)	Forecast	Forecast error (%)	Forecast	Forecast error (%)
2021/7/1	7.14	13.09	83.33	7.11	-0.42	8.48	18.77
2021/8/1	7.72	7.19	-6.87	6.36	-17.62	7.12	-7.77
2021/9/1	16.61	11.63	-29.98	6.75	-59.36	8.74	-47.38
2021/10/1	15.28	11.65	-23.76	7.9	-48.3	8.94	-41.49
2021/11/1	17.03	15.19	-10.8	13.24	-22.25	11.86	-30.36
2021/12/1	4.98	2.46	-50.6	2.09	-58.03	1.75	-64.86
2022/1/1	1.08	1.43	32.41	1.33	23.15	0.94	-12.96
2022/2/1	1.49	2.37	59.06	1.47	-1.34	1.15	-22.82
2022/3/1	3.73	2.4	-35.66	2.19	-41.29	2	-46.38
2022/4/1	2.15	3.45	60.47	2.5	16.28	2.17	0.93

Note: ETS, Exponential Smoothing; SARIMA, Seasonal Autoregressive Integrated Moving Average.

## Discussion

As can been seen from the descriptive statistics in Table 1 and the seasonal distribution of FBD in Fig. 1 that the number of reported cases from December 2019 to April 2020 was at low level. Possibly due to the outbreak of COVID-19, In response to the outbreak of the COVID-19 in December 2019, by the end of February, China’s national, provincial and municipal governments had taken a series of public health interventions to effectively curb the epidemic [31], such as lockdown measures. People are staying at home rather than taking the risk of going to hospital, so there are far fewer reported cases of FBD.

We analyzed the FBD incidence rates and observed a fluctuating downward trend and seasonal characteristics in this study, with the valley is in January to February, and the incidence is higher from June to November almost every year. The reasons could be as follows: In the first place, Chinese New Year usually falls in January or February, and it is traditionally considered bad luck to go to the hospital at this time, resulting the reduction of reported incidence case [32]. In the second place, higher temperatures in June, July and August make food spoil more quickly, which is more likely to cause FBD. Besides, this phenomenon may be related to the climate and human behaviors [33], as temperatures rise, social activities and contacts between people tend to increase during the spring and summer months. Thus, the reported incidence is consistently high during the three months of each year, leading to the widespread epidemics. In addition to this, from Fig. 1 we can see that September to November are also high incidence month of FBD, studies have shown that cold weather or wind can affect the incidence of some diseases [34].

Many scholars have studied the disease prediction model. Some research shows that the ARIMA model is suitable for complex interactions between temporal seasonal effects, long-term trends, and random fluctuations. This model is one of the commonly used time series analysis models for predicting infectious diseases, such as tuberculosis, hand-foot-mouth disease, mumps, influenza etc. [35–38]. What’s more, the “STL” function is used to decompose the series, which can not only display the trend and seasonal change of the incidence rate series of FBD, but also calculate the seasonal index of each month, which can intuitively understand its seasonality [39]. We use the SARIMA model to perform linear fitting on the FBD series. By comparing the AIC, BIC values, SARIMA(1, 0, 0)× (1, 1, 0) _12_ is the best model, and the RMSE and MAPE values of this model are 4.23 and 28.80, respectively. Holt-Winters model has high prediction accuracy the periodic regularity disease. Besides, ETS model take comprehensive historical information into consideration, it’s also a good method. In order to highlight the performance accuracy of developed SARIMA, ETS, and Holt-Winters models, we divided the FBD time series sample into two parts. The first part of data, June 2017 to June 2021, as a training set for in-sample simulated modeling. The rest of 10 month, July 2021 to April 2022, as a testing set. Based on these modellings’ accuracy metrics, it can be seen from the evaluation indicators of the three models (Table 3), we found the MSE, MAE, RMSE of the Holt-Winters model are 8.78, 2.33 and 2.96 respectively. It’s indicated the Holt-Winters model has better predictive accuracy than SARIMA and ETS models. This may be due to the different characteristics of each model, as the Holt-Winters model is built using historical data and does not consider the interference of other factors [32]. Therefore, this model has certain reference value for predicting FBD. And through Fig. 2, we can also clearly see that the fitting curve of the Holt-Winters model is closer to the true value.

At present, FBD hamper socio-economic development by placing pressure on health care systems and harming national economies, tourism and trade [40]. Despite significant improvements in medical services and infectious disease control capabilities, FBD still remain a major public health problem in China [41]. Like other countries, FBD characterized by acute gastrointestinal diseases are the largest food safety issue and the most disturbing public health threat related to food in China [42, 43]. However, few studies have chosen the optimal model by comparing the degree of fit of the three models. The purpose of this study is to select the model with the best prediction and fitting performance by comparing several common models, in order to better assist in the rational allocation of medical resources and personnel, and provide clues for the prevention and treatment of this disease from data analysis. Reliable prediction of FBD helps to better coordinate the relief and intervention resources of the public health system, and alleviate the pressure on the healthcare system [44]. In addition, the optimal prediction model of FBD obtained in this study can also be used in other countries and regions to predict and control this disease.

Although we have selected the optimal prediction model for FBD by comparing the three, there are still some shortcomings that need to be improved. In this study, we chose the model with good overall forecasting effect, but the performance of the three models is various in different months, so the month-specific forecasting model may need further research and verification in the future. Besides, the data is not comprehensive enough, and we should consider combining more years of data to make better predictions in future research.

## Conclusions

We used SARIMA model, Holt-Winters model and ETS model in predicting the incidence of FBD, from which we found the highest incidence was November 2021and the lowest incidence occurred in January 2019. The incidence of FBD presented obvious seasonal trends in this study. By comparing of prediction results of three models, in most months of the forecast, the forecast error of the Holt-Winters model was smaller than that of the other two models. When make comparison of the fitting effects between the three models, the fitting value of the Holt-Winters model is closer to the actual value. Therefore, it can be concluded that the optimal model is Holt-Winters model, which can provide convenience and new ideas for related forecasting research in the future.

## Data Availability

The datasets analyzed during the current study are not publicly available due to the data being investigated and protected by the Chongqing Nan’an District Center for Disease Prevention and Control, but are available from the corresponding author on reasonable request.

## Abbreviations

SARIMA: Seasonal autoregressive integrated moving average
FBD: Foodborne disease
ETS model: Exponential Smoothing model
WHO: World Health Organization
ADF: Augmented Dickey Fuller
ACF: Autocorrelation functions
PACF: Partial autocorrelation functions
AIC: Akaike information criterion
BIC: Bayesian information criterion
RMSE: Root means square error
MAE: Mean absolute error MAE
MAPE: Mean absolute percentage error
MSE: Means square error
AR: Autoregressive
MA: Moving average
